# Diagnostic accuracy of calculated serum osmolarity to predict dehydration in older people: adding value to pathology laboratory reports

**DOI:** 10.1136/bmjopen-2015-008846

**Published:** 2015-10-21

**Authors:** Lee Hooper, Asmaa Abdelhamid, Adam Ali, Diane K Bunn, Amy Jennings, W Garry John, Susan Kerry, Gregor Lindner, Carmen A Pfortmueller, Fredrik Sjöstrand, Neil P Walsh, Susan J Fairweather-Tait, John F Potter, Paul R Hunter, Lee Shepstone

**Affiliations:** 1 Norwich Medical School, University of East Anglia, Norwich, UK; 2 Norfolk and Norwich University Hospitals NHS Foundation Trust, Norwich, UK; 3 Department of General Internal Medicine, Inselspital, University Hospital Bern, Bern, Switzerland; 4 Department of General Anesthesiology, Intensive Care and Pain Management, Medical University of Vienna, Vienna, Austria; 5 Department of Emergency Medicine, Södersjukhuset AB, Stockholm, Sweden; 6 College of Health and Behavioural Sciences, Bangor University, Bangor, UK

**Keywords:** GERIATRIC MEDICINE, PREVENTIVE MEDICINE, NUTRITION & DIETETICS

## Abstract

**Objectives:**

To assess which osmolarity equation best predicts directly measured serum/plasma osmolality and whether its use could add value to routine blood test results through screening for dehydration in older people.

**Design:**

Diagnostic accuracy study.

**Participants:**

Older people (≥65 years) in 5 cohorts: Dietary Strategies for Healthy Ageing in Europe (NU-AGE, living in the community), Dehydration Recognition In our Elders (DRIE, living in residential care), Fortes (admitted to acute medical care), Sjöstrand (emergency room) or Pfortmueller cohorts (hospitalised with liver cirrhosis).

**Reference standard for hydration status:**

Directly measured serum/plasma osmolality: current dehydration (serum osmolality >300 mOsm/kg), impending/current dehydration (≥295 mOsm/kg).

**Index tests:**

39 osmolarity equations calculated using serum indices from the same blood draw as directly measured osmolality.

**Results:**

Across 5 cohorts 595 older people were included, of whom 19% were dehydrated (directly measured osmolality >300 mOsm/kg). Of 39 osmolarity equations, 5 showed reasonable agreement with directly measured osmolality and 3 had good predictive accuracy in subgroups with diabetes and poor renal function. Two equations were characterised by narrower limits of agreement, low levels of differential bias and good diagnostic accuracy in receiver operating characteristic plots (areas under the curve >0.8). The best equation was osmolarity=1.86×(Na^+^+ K^+^)+1.15×glucose+urea+14 (all measured in mmol/L). It appeared useful in people aged ≥65 years with and without diabetes, poor renal function, dehydration, in men and women, with a range of ages, health, cognitive and functional status.

**Conclusions:**

Some commonly used osmolarity equations work poorly, and should not be used. Given costs and prevalence of dehydration in older people we suggest use of the best formula by pathology laboratories using a cutpoint of 295 mOsm/L (sensitivity 85%, specificity 59%), to report dehydration risk opportunistically when serum glucose, urea and electrolytes are measured for other reasons in older adults.

**Trial registration numbers:**

DRIE: Research Register for Social Care, 122273; NU-AGE: ClinicalTrials.gov NCT01754012.

**Strengths and limitations of this study**

- Dehydration has become a generic term – we have clearly described dehydration type (water-loss dehydration) and used serum osmolality, the correct reference standard.
- Assessment of equations in five different groups of older people including healthy free-living older people, frailer people living in residential care, and older people visiting emergency care or staying in hospital, living in several European countries, and including men and women, people with and without poor renal function, people with and without diabetes, with and without dehydration.
- Careful measurement of osmolality and the components of osmolarity in hospital laboratories with good standardisation or under research conditions, from community and hospital samples.
- Lack of incorporation of alcohol into the equations, though we did assess the effect of mild inebriation and found it only modestly affected results.

## Background

Twenty per cent of older people living in UK residential care are dehydrated,^1^ 40% dehydrated on UK hospital admission^2^ and >20% of free-living US older people^3^ ^4^ are dehydrated. Despite increased risks of disability, mortality and hospital admissions associated with water-loss dehydration (or simply dehydration, not to be confused with hypovolaemia, see box 1 for definitions)^5–7^ dehydration is often unnoticed. Our aim was to add value to routine blood test results by using them to screen for dehydration in older people.

Box 1 **Key concepts and definitions**

*Dehydration*: water-loss dehydration, the result of insufficient fluid intake, which leads to elevation of directly measured serum osmolality.

*Directly measured serum osmolality*: the osmotic concentration of blood serum, expressed as the number of milliosmoles of solute per kilogram of serum water.^8^ Directly measured serum osmolality is assessed by degree of freezing point depression. Normal values for directly measured serum osmolality, indicating euhydration, are 275 to ≤295 mOsm/kg, while 295 to 300 mOsm/kg is classified as impending dehydration, and >300 mOsm/kg as current dehydration.^9^

*Directly measured plasma osmolality*: the osmotic concentration of blood plasma, expressed as the number of milliosmoles of solute per kilogram of plasma water. Directly measured plasma osmolality is assessed by degree of freezing point depression. Thought to be equivalent to directly measured serum osmolality.

*Calculated serum osmolarity*: is an estimation of the osmolar concentration of serum and is proportional to the number of particles *per litre of solution*; it is expressed as mOsm/L. This is what is used when a calculated value is derived (for further details see Deardorff^8^).

Water-loss dehydration is due to insufficient fluid intake and is characterised by an increase in directly measured (by freezing point depression) serum/plasma osmolality. Directly measured osmolality is the reference standard for hydration status in older people because osmolality is central to physiological fluid control, acting as a trigger for thirst and renal fluid conservation,^10^ a single measurement can diagnose dehydration (without prior information), and other measures do not work well in older people.^9^ ^11^ ^12^ Serum urea/creatinine ratio is non-specific and not useful in indicating hydration status when kidneys are not functioning well, common in older adults.^9^ ^13^ While low fluid intake is indicated in young adults by alterations in urinary parameters,^14^ ^15^ actual dehydration appears to be better characterised even in young adults by plasma or serum osmolality,^12^ and in older adults urinary measures do not indicate hydration status as poor urinary concentration is common^16^ ^17^ (L Hooper, DK Bunn, A Abdelhamid, *et al.* Dehydration assessed using urinary tests, how well do they work? Diagnostic accuracy in older people. *Am J Clin Nutr* 2015; submitted). Weight fluctuates in well-hydrated older people and dehydration may occur gradually rather than acutely, so sudden weight loss is not a good indicator.^18^ ^19^ Physician assessment of hydration status is not consistent between physicians,^20^ and bioelectrical impedance does not appear accurate enough.^21–23^ For these reasons serum and plasma osmolality are stated as the reference standards for diagnosing water-loss dehydration in older adults.^9^ ^12^ ^24^ ^25^

Monitoring directly measured serum osmolality will provide information on hydration status, but would be invasive, and is only partially automated in UK pathology laboratories so is expensive–growing numbers of requests for directly measured serum osmolality would cause sample handling problems in the clinical laboratory. Simple tests such as urine measures and skin turgor have not shown promise in screening for dehydration in older people^16^ ^17^ (L Hooper, *et al*. 2015, submitted), and calculated osmolarity is recommended in standard medical textbooks and doctors’ websites to assess for dehydration.^26–29^ Components of osmolarity equations (sodium, potassium, urea and glucose) are commonly measured as part of routine blood tests when older people visit hospitals or general practitioners. Many osmolarity equations have been developed and are in use, but it is not clear which are most useful. In the Dehydration Recognition In our Elders (DRIE) study, which included older people living in UK residential care, we assessed diagnostic accuracy of different calculated osmolarity formulae, compared to directly measured serum osmolality and identified an osmolarity formula usefully diagnostic for dehydration.^1^ Calculated osmolarity >296 mOsm/L had high sensitivity (97%) and reasonable specificity (76%), and a diagnostic OR of 99. A calculated osmolarity equation that accurately identifies dehydration in the full spectrum of older people (healthy older people, frailer people in residential care and those needing secondary care) could enable pathology laboratories to use routine blood tests to provide individual information on hydration status. This would enable health professionals to support older people to maintain or increase their fluid intake. We assessed which osmolarity equation best predicts directly measured serum/plasma osmolality (and dehydration) in five cohorts of older people, and whether it could be used to add value to routine blood test results through screening for dehydration in older people.

## Methods

### Datasets

We assessed osmolarity equations in five data sets, each of which assessed directly measured osmolality, sodium, potassium, glucose and urea from a single blood draw for each participant. For each data set we removed participants aged <65 years, those missing any of serum/plasma osmolality, serum sodium, potassium, urea or glucose measurements or who presented values resulting from artefact or physiological extremes (potassium >8 mmol/L, sodium <80 mmol/L, osmolality >340 mOsm/kg). Estimated-glomerular filtration rate (eGFR) was calculated with the Modification of Diet in Renal Disease formula,^30^ truncated at 90 to reflect clinical practice. The reference standard was directly measured serum/plasma osmolality categorised as hydrated (275 to <295 mOsm/kg), impending dehydration (295–300 mOsm/kg) or current dehydration (>300 mOsm/kg).^9^ ^19^

The Dietary Strategies for Healthy Ageing in Europe (NU-AGE) study was a randomised controlled multicentre trial of healthy, independent older people (without frailty, heart failure or serious chronic illness) responsible for their own shopping/cooking/meal choice and preparation aged 65–79 years (http://www.nu-age.eu). The NU-AGE project aimed to assess effects of a 1 year dietary intervention on markers of inflammation and health.^11^ ^31^ ^32^ We used cross-sectional baseline data from 271 Norfolk (UK) participants recruited between September 2012 and January 2014, of whom 238 had measured serum osmolality. Two were excluded (one potassium >8 mmol/L, one missing serum glucose), so 236 were included in analyses. Participants were asked to avoid alcohol for 24 h before phlebotomy, but this was not verified. Venepuncture was in the morning after ≥8 h fasting (though participants were encouraged to drink water). Whole blood was processed (using clot activator tubes) to give serum samples, and stored at −80°C until analyses. Samples were analysed in the Department of Clinical Biochemistry, Norfolk and Norwich University Hospital (NNUH, Norfolk, UK). The laboratory is fully accredited (Clinical Pathology Accreditation), performs daily calibration, internal quality control and participates in External Quality Assessment. Serum osmolality was measured by depression of freezing point (Advanced model 2020 multisample osmometer; Advance Instruments, repeatability of±3 mOsm/kg, SD 1, in the 0–400 mOsm/kg region, coefficient of variance (CV) as for DRIE samples, 0.6%), frozen samples defrosted at room temperature on a roller mixer. When sufficient blood was available we also assessed serum sodium and potassium (indirect ion-selective electrode, ISE; Abbott Architect), urea (using urease; Abbott Architect), creatinine (enzymatic method; Abbott Architect), haemoglobin (Sysmex XN) and glucose (hexokinase/glucose 6-phosphate dehydrogenase; Abbott Architect). It is not known whether laboratory staff analysing directly measured serum osmolality were aware of other blood measurements or vice versa.

The DRIE cohort were aged ≥65 years (range 65–105) living in residential care in Norfolk and Suffolk (UK), with a variety of cognitive and/or functional impairments. Those with heart failure, end-stage renal failure or terminal illness were excluded.^1^ ^33^ Recruitment occurred between April 2012 and August 2013, and this analysis used baseline (cross-sectional) data. Full details of recruitment criteria, consent and the study flow have been published previously.^1^ Participants had low levels of self-reported alcohol intake, and none appeared inebriated on interview. During the interview non-fasting venous blood samples were collected from an antecubital vein or back of hand using needle and syringe after participants had rested sitting (occasionally lying) ≥5 min. Samples were immediately transferred to SST vacutainers, stored at room temperature, delivered to the Department of Clinical Biochemistry (NNUH) within 4 h and analysed immediately for serum osmolality. Serum analyses for DRIE were as for NU-AGE (same laboratory, personnel, accuracy and equipment). We sent 19 hidden duplicate samples for serum osmolality analysis to the NNUH laboratory between June 2014 and January 2015 (samples taken from the same blood draw, but in separate tubes labelled with different sample numbers) to assess CV. The laboratory mean CV for these 19 duplicates was 0.6%. Of 201 people living in residential care recruited and interviewed, 198 had directly measured serum osmolality, of whom 26 were missing serum glucose, so 172 were included in this analysis.

Fortes included people aged ≥60 years admitted to Welsh acute medical care or emergency departments with any primary diagnosis and capacity to consent May–November 2011.^17^ Those too unwell, who had begun medical treatment or rehydration were excluded. Blood was collected from antecubital or dorsal metacarpal veins without venestasis into one lithium heparin coated vacutainer (Becton Dickinson, Oxford, UK) and centrifuged immediately (1500*g*, 10 min, 4°C). Plasma was aspirated and directly measured plasma osmolality assessed (freezing point depression osmometer, Model 330 MO; Advanced Instruments, Norwood, Massachusetts, USA) in duplicate. Where the difference was <3 mOsm/kg the mean was used, otherwise further repeats were carried out until the mean was clear. The mean CV for the 2–8 duplications for each sample (mean 2.8 duplications) was 0.7%. Standard solutions (290 mOsm/kg) were run daily to ensure±2 mOsm/kg precision. Serum sodium, potassium, urea and glucose were analysed in the hospital clinical biochemistry department (indirect ISE, Olympus AU 2700 automated chemistry immuno-analyser; Beckman Coulter, Brea, California, USA), so analysis of osmolarity components was blind to directly measured osmolality and vice versa. Participants were not asked about recent alcohol intake, but those clearly inebriated could not give informed consent. Of 180 participants recruited, one did not have plasma osmolality measured, 10 lacked sodium, 62 glucose and 10 were <65 years, leaving 97 analysed.

Sjöstrand recruited older adults (≥75 years) able to provide informed consent and not critically ill who attended the emergency room of a Swedish tertiary care centre in spring-summer 2010. Those taking ACE inhibitors, >40 mg/day diuretics or >50 mg/d β-blockers were excluded, as were those with heart failure or under the influence of alcohol (assessed by study nurse, not discussed or tested). Main study results on fluid dynamics over several hours are not published, but some aspects have been reported.^16^ ^34^ This analysis used baseline directly measured serum osmolality and serum measures. Serum samples were analysed immediately at the Karolinska ISO-certified laboratory, osmolality measured using freezing point depression (Osmometer Advanced 2020, Advanced Instruments Inc, USA, CV unclear), serum sodium, potassium, urea, creatinine and glucose assessed (by indirect ISE, glucose assessment was duplicated and mean recorded, Hitachi 917, Naka, Japan). The osmometer was automated, so assessment of serum osmolality was independent of other blood measures. Of 41 older adults recruited, 5 were excluded (one had serum osmolality >340 mOsm/kg, two were missing potassium values and two urea), so 36 were included here.

Pfortmueller included adults admitted to a Swiss emergency department with primary diagnosis of decompensated liver cirrhosis, January 2002 to December 2012.^35^ Pfortmueller's retrospective analysis aimed to assess the association of glucose disturbances with outcome, and the select group included a high proportion with very low or raised non-fasting glucose. Patients were identified via computerised patient database (Qualicare Office, Medical Database Software; Qualidoc AG, Bern). Directly measured serum osmolality (Advanced 3900 osmometer, assessment of freezing point depression, CV <1%) and electrolytes, urea and glucose (indirect ISE, Roche Modular 800 System) were measured by the Department of Clinical Chemistry, Bern University Hospital. Thirty-one participants were alcoholic, but recent alcohol intake was not recorded. It is unclear whether laboratory staff analysing directly measured serum osmolality were aware of other blood measures or vice versa. Of 312 participants in the data set, 58 were ≥65 years, of whom we excluded four (one with osmolality >340 mOsm/kg, three potassium >8 mmol/L), therefore 54 were included in analyses.

### Osmolarity equations

Fazekas *et al*^36^ collected 36 equations to calculate osmolarity. Since sodium, potassium, glucose and urea are regularly measured in older people undergoing blood tests, we focused on the 33 equations that only included these factors (omitting 3 equations including ionised calcium or lactate)^37^ ^38^ (V A Nelson, R A Scheidt. Personal communication to Fazekas *et al* 2013, 1969). Fazekas multiplied results of several equations by 0.985 (as they were reported in mOsm/L^39–41^); however, as this may not have been the original authors’ intention we ran these equations with and without this multiplication (adding equations 25a and 27a). We also evaluated equations we have observed local physicians using including the Wikipedia equation,^26^ US National Health and Nutrition Examination Survey,^42^ MDcalc and Joint British Diabetes Societies equations^27^ ^29^ and tonicity (associated with adverse outcomes^6^) (see online supplementary table S1). We assessed equivalence of each of the 39 calculated osmolarity equations to the reference standard.

### Terminology and units

Directly measured osmolality was assessed in molal units (mOsm/kg) and calculated osmolarity produced molar units (mOsm/L) making terminology comparing the two measurements complex. For clarity we have written all equations using SI units, referred to as calculated osmolarity, and expressed in mOsm/L. Directly measured osmolality was measured and reported in mOsm/kg, while serum sodium, potassium, urea and glucose measurements were in mmol/L. Since we were aiming for equivalence between osmolarity and osmolality, units for the osmolar gap, the difference between directly measured osmolality and calculated osmolarity, were labelled mOsm.^43^

### Statistical analysis

We used descriptive statistics to summarise each cohort, and Pearson's correlation to assess associations between directly measured osmolality and serum sodium, potassium, urea, creatinine and glucose. Osmolarity was calculated using each of the 39 formulae for each participant and compared against directly measured osmolality (difference in mOsm, measured osmolality in mOsm/kg minus calculated osmolarity in mOsm/L), then averaged for each equation in each cohort. We were interested in osmolarity equations which approximated directly measured osmolality, so we identified equations where:

1. Mean difference was −1 to+1 mOsm;
2. There was no statistically significant difference between osmolarity equation results and directly measured osmolality (p value for the paired t test ≥0.01, set at 0.01 due to multiple testing and aiming not to lose potentially useful equations at the beginning of the selection process).

We were interested in equations where ≥3 of the 5 cohorts fulfilled either of these criteria.

Having chosen the five most useful equations this way, remaining analyses used only these equations. We assessed percentage of participants whose osmolarity equation results fell within 2% of directly measured osmolality for each cohort and used a weighted mean to assess equations across all cohorts and for specific subgroups. Bland-Altman plots compared each osmolarity equation with directly measured osmolality, plotting the difference against the mean of osmolality and osmolarity. To assess differential bias, Pearson’s correlation assessed the association of the difference with osmolality, biochemical parameters (haemoglobin, sodium, potassium, glucose, urea and eGFR), age and measures of nutritional, cognitive and functional status. We created receiver operating characteristic (ROC) plots to compare the ability of each of the five equations to diagnose current dehydration (serum/plasma osmolality >300 mOsm/kg). Sensitivity and specificity, positive and negative likelihood ratios were calculated for each equation compared to current dehydration, assessing specificity where sensitivity was ≥75%, ≥80%, ≥85% and ≥90%, and assessing the sensitivity and specificity of an appropriate whole-number cutpoint. A decision threshold was determined using the method of Zweig and Campbell,^44^ calculating a slope m=(false-positive cost/ false-negative cost)×(1-dehydration prevalence)/(dehydration prevalence). The best decision threshold was the point on the ROC curve where the line with this slope was tangent. All statistical analyses were carried out in excel or in STATA (IC 11.2), and statistical significance set at p<0.05 unless otherwise stated. This paper conforms to STARD reporting standards for diagnostic studies.^45^

## Results

Participants’ are characterised by cohort in table 1. Briefly, NU-AGE participants (mean age 70 years) had good cognitive and functional status, and few participants had raised sodium, potassium, glucose or low eGFR. DRIE care home residents were older (mean 86 years), with lower cognitive and functional status, >40% had poor renal function (eGFR<60), 20% raised glucose. Pfortmueller participants were relatively young (mean 69 years, similar to NU-AGE), with high proportions of hyperglycaemia and hyperkalaemia, while Sjöstrand emergency department participants were older (mean 84 years, similar to DRIE) with high levels of dehydration and hypernatraemia. Fortes participants (mean 79 years) had the lowest levels of dehydration, but 36% had poor renal function. Current dehydration (directly measured serum/plasma osmolality >300 mOsm/kg) varied from 8% of participants (Fortes) to 44% (Sjöstrand).

**Table 1:** Descriptive characteristics of participants of the five cohorts

Type of older participants	NU-AGE (n=236)	DRIE (n=172)	Fortes (n=97)	Sjöstrand ED (n=36)	Pfortmueller (n=54)
	Free-living, healthy	Residential care, frailer	Admitted to medical care /emergency department	Awaiting emergency room treatment	Decompensated liver cirrhosis
Age, years (all ≥65 years)	70.1 (4.1)	86.0 (7.9)	78.6 (7.5)	83.8 (5.9)	69.3 (4.3)
Sex, n (%) female	147 (62%)	111 (65%)	50 (52%)	20 (56%)	13 (24%)
Weight, kg	74.3 (13.8)	69.5 (17.3)	ND	68.4 (13.4)	ND
Height, cm	166.0 (8.8)	163.6 (10.6)	ND	169.7 (9.1)	ND
BMI, kg/m^2^	26.9 (4.1)	25.9 (5.6)	ND	24.0 (4.4)	ND
MMSE	28.4 (1.5)	22.2 (5.6)	ND	ND	ND
Functional status*	IADL 6.8 (1.5)	BI 67.3 (26.4)	ND	ND	ND
Serum osmolality, mOsm/kg	296.0 (7.0)	291.9 (9.5)	286.7 (14.4)	299.7 (7.0)	290.9 (8.6)
Osmolality, n (%) >300 mOsm/kg	53 (22%)	33 (19%)	8 (8%)	16 (44%)	6 (11%)
Sodium, mmol/L	140.7 (2.2)	137.4 (3.9)	136.6 (5.2)	142.6 (2.3)	135.6 (4.4)
Sodium, n (%) >145 mmol/L	1 (<1%)	1 (1%)	2 (2%)	3 (8%)	1 (2%)
Potassium, mmol/L	4.3 (0.3)	4.2 (0.4)	4.4 (0.6)	3.9 (0.4)	4.3 (0.9)
Potassium, n (%) >5.0 mmol/L	2 (1%)	6 (3%)	10 (10%)	0 (0%)	9 (17%)
Urea, mmol/L	5.4 (1.2)	7.0 (2.7)	8.8 (6.1)	8.2 (2.8)	9.7 (6.2)
Creatinine, µmol/L	79.6 (15.7)	90.2 (36.3)	118.9 (76.4)	84.0 (28.8)	123.0 (91.6)
Glucose, mmol/L	5.2 (0.7)	6.9 (3.2)	7.1 (2.6)	5.7 (1.2)	6.7 (4.0)
Glucose, n (%) >7.8 mmol/L	2 (1%)	34 (20%)	25 (26%)	1 (3%)	19 (35%)
Diabetes diagnosed	6 (3%)	32 (19%)	19 (20%)	ND	27 (50%)
eGFR, mL/min†	75.0 (10.9)	63.7 (19.1)	57.2 (19.3)	71.5 (17.3)	63.9 (24.4)
Poor renal function, n (%) eGFR 30 to <60	23 (10%)	65 (38%)	36 (37%)	13 (36%)	14 (26%)
eGFR <30, n (%)	0 (0%)	7 (4%)	11 (11%)	0 (0%)	6 (11%)
Haemoglobin, g/dL	13.9 (1.0) n=231	12.4 (1.5)	12.4 (2.2)	13.0 (1.5)	ND

All numbers are mean (SD) except where otherwise labelled.

*IADL (Instrumental Activities of Daily Living) and BI (Barthel Index) assess functional status. IADL scores from 0 to 8 and 8 is fully functioning, while the BI scores from 0 to 100, with 100 being fully functioning.

†For creatinine in µmol/L the following equation was used, and truncated at 90 (top measure)^30^:
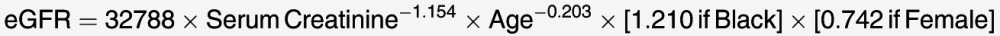

BMI, body mass index; eGFR, estimated-glomerular filtration rate; MMSE, Mini-Mental State Examination; ND, no data (not measured).

Adverse events associated with blood draws include bruising, but no cohorts recorded bruising or noted other adverse effects of participation.

In all cohorts apart from Pfortmueller, sodium concentrations were strongly, statistically significantly correlated with serum/plasma osmolality (see online supplementary table S2). Pfortmueller data showed no statistically significant relationships between sodium, potassium, urea, creatinine or glucose and directly measured osmolality. Urea was less strongly but significantly correlated with directly measured osmolality. Potassium and creatinine were weakly and significantly or borderline significantly correlated with directly measured osmolality in NU-AGE, DRIE and Fortes, but not Sjöstrand or Pfortmueller. Glucose was weakly and significantly correlated with directly measured serum osmolality in DRIE, but not significantly in other cohorts. This suggested that useful osmolarity equations would probably include sodium, urea or creatinine and probably potassium and glucose. Participants with raised serum sodium, potassium, urea and glucose were classified by hydration status in online supplementary table S3.

### Absolute bias

The difference (directly measured osmolality, mOsm/kg, minus calculated osmolarity, mOsm/L) varied from −37.6 mOsm (Fortes equation 27) to 31.8 mOsm (NU-AGE equation 1). The p values (paired t test) comparing osmolarity and osmolality are displayed in online supplementary table S4. We were interested in equations where for ≥3 of 5 cohorts the mean difference was −1 to+1 or the p value was ≥0.01. Equations which fulfilled the first criterion were 10, 24, 32 and 33, while equations 10, 24, 26, 32 and 33 fulfilled the second and were examined further.

### Predictive accuracy

For these five equations we calculated percentage of participants whose osmolarity equation results fell within 2% of directly measured osmolality (see online supplementary figure S1). Percentages were 70–90% for most equations for NU-AGE, DRIE and Sjöstrand, but lower in Fortes (40–50%) and Pfortmueller (30–50%). We created a weighted mean percentage for each equation across all five cohorts, which confirmed equation 32 as consistently useful and returning a greater proportion of participants within 2% of directly measured osmolality.

**Figure 1:**
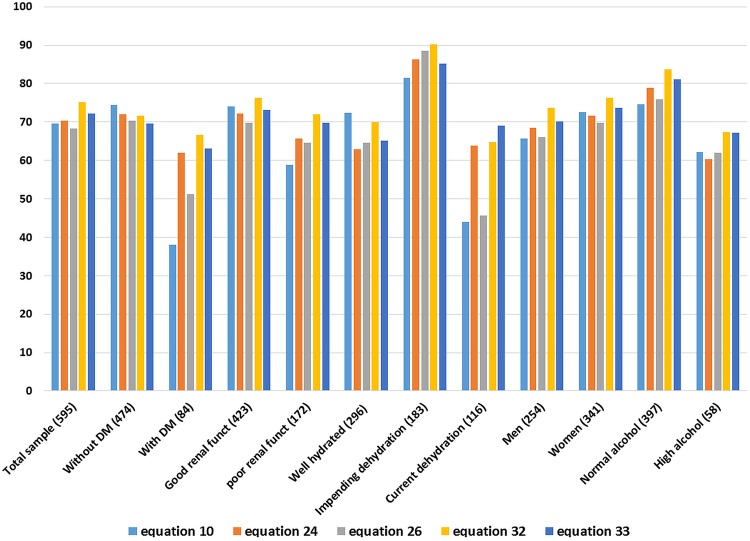
Percentages of individuals whose calculated osmolarity fell within 2% of measured osmolality, by equation and by specific conditions*. DM: diabetes mellitus. *In assessing by alcohol intake we had limited information on recent alcohol intake in any cohort, but Dehydration Recognition In our Elders (DRIE) participants reported very low alcohol intake, and the Dietary Strategies for Healthy Ageing in Europe (NU-AGE) participants had usual alcohol intake assessments so we separated out those who drank ≥21 g alcohol/week (intake mean plus one SD). For Pfortmueller we separated out alcoholics, other cohorts were not represented.

Predictive accuracy was assessed for specific subgroups: people with and without diagnosed diabetes mellitus; with good or poor (eGFR <60) renal function; with normal hydration, impending or current dehydration; men and women; low-alcohol and high-alcohol intake (figure 1). Equations 10 (which did not include glucose) and 26 performed less well than other equations where participants were diabetic or had current dehydration. Equation 32 returned a higher proportion of participants within 2% of directly measured osmolality than the other equations for all subgroups, except of those who were without diabetes, well hydrated and currently dehydrated (when it was second most predictive).

### Bland-Altman analysis

Bland-Altman plots compared the five osmolarity equations with directly measured osmolality,^46^ with 95% limits of agreement (see online supplementary figure S2). Narrower limits imply better agreement, and smaller mean difference suggests near equality of osmolality and osmolarity. The narrowest limits were provided by formula 32 for NU-AGE, DRIE and Fortes, and formula 32 was a close second to formula 33 for Sjöstrand, though not good for Pfortmueller. Equation 10 did not show the narrowest limits for any data set, equation 24 was joint equal for NU-AGE only, and equation 26 was narrowest for Pfortmueller.

### Differential bias

For all five equations in all data sets the difference was positively associated with directly measured osmolality, although correlations were less strong for equations 32 and 33 (see online supplementary table S5). There was a tendency for the difference in equations 10, 24 and 26 to be significantly associated with sodium, potassium, urea, creatinine, glucose and eGFR values in several data sets (DRIE and Fortes in particular) while equations 32 and 33 appeared to be less related. No equations were consistently associated with age, body mass index, haemoglobin, cognitive or functional status.

### Diagnostic accuracy

**Figure 2:**
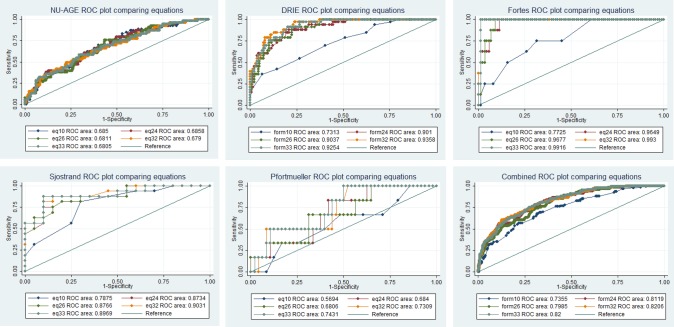
ROC plots for each equation for each data set and for all data sets combined. DRIE, Dehydration Recognition In our Elders; NU-AGE, the Dietary Strategies for Healthy Ageing in Europe; ROC, receiver operating characteristic.

ROC plots compared ability of each equation to diagnose current dehydration (serum/plasma osmolality >300 mOsm/kg) (figure 2, see online supplementary table S6). Diagnostic accuracy is represented by area under the curve (AUC, greater AUC equating to greater diagnostic accuracy, maximum 1.0). Equation 32 showed greatest ROC_AUC_ in DRIE, Fortes and Sjöstrand, equation 24 was more useful in NU-AGE and 33 in Pfortmueller. In the combined data set (595 participants) equations 32 and 33 had similar diagnostic accuracy (ROC_AUC_ 0.821 and 0.820, respectively). In the combined data sets omitting Pfortmueller, which could be said to be atypical, had little effect on diagnostic accuracy and equations 32 and 33 were still preferable (ROC_AUC_ 0.831 and 0.828, respectively, data not shown). Ensuring sensitivity ≥75% specificity was 71% for equation 32 and 73% for equation 33. Raising sensitivity to ≥80% produced specificity of 67% for both equations (see online supplementary table S7). At a cutpoint of ≥296 for equation 32, sensitivity for current dehydration was 80% and specificity 66%, positive likelihood ratio 2.36, and negative likelihood ratio 0.30. At a cutpoint of ≥297 for equation 33, sensitivity for current dehydration was 78% and specificity 69%, positive likelihood ratio 2.54 and negative likelihood ratio 0.31.

### Overall diagnostic accuracy

Table 2 summarises the performance of the five equations across the characteristics we tested. Equations 32 and 33 performed well across all assessments, but equation 32 was consistently most useful.

**Table 2:** Summary of results of analyses by equation

Test	Eq10	Eq24	Eq26	Eq32	Eq33
Percentage of participants with osmolarity within 2% of osmolality	X	–	X	☼	–
Bland-Altman analyses	X	−	−	☼	−
Differential bias	X	X	X	☼	☼
ROC plots	X	−	X	☼	☼
Sensitivity and specificity	X	−	−	☼	☼

X, indicates that a test is not useful; –, indicates a test which sometimes appears useful, but not consistently; ☼, that it does well and appears particularly useful.

ROC, receiver operating characteristic.

Using the ROC plot for equation 32 (figure 2, combined, see yellow line) we chose a decision threshold for dehydration as assessed by equation 32.^44^ The mean prevalence of dehydration across all five cohorts was 0.19, and we estimated that the cost of a false-negative finding (missing that a person is dehydrated, with its health consequences) has five-times the cost of a false-positive finding (labelling a person as dehydrated when they are not, resulting in a further blood test to directly measure osmolality or simply encouraging them to drink more). This gave a slope of 0.2×0.81/0.19=0.85. A line with a slope of 0.85, is tangent to the equation 32 ROC curve at the cutpoint of 295 mOsm/L giving sensitivity of 84.5% and specificity of 58.9% (with a positive likelihood ratio of 2.05 and a negative likelihood ratio of 0.26).

## Discussion

We report the most comprehensive assessment of the relationship between calculated osmolarity and directly measured osmolality in older people to-date. One osmolarity equation (equation 32, by Khajuria and Krahn^47^) estimated directly measured serum/plasma osmolality well across healthy and frail older people, those in and out of hospital, with and without diabetes, with and without poor renal function, at all levels of directly measured serum osmolality (or dehydration) and in men and women. This equation (osmolarity=1.86×(Na^+^+K^+^)+1.15×glucose+urea+14) had ROC_AUC_ 0.82 (95% CI 0.78 to 0.86). Using an osmolarity cutpoint of >295 mOsm/L gives sensitivity of 85% accompanied by specificity of 59% over the full range of older participants. Of the other, widely used, osmolarity equations (including equations suggested by standard medical sources) many were poor at predicting directly measured serum/plasma osmolality, the reference standard for water-loss dehydration in older people. This suggests that some equations should not be used to estimate directly measured osmolality.

Study strengths include careful measurement of osmolality and the components of osmolarity in hospital laboratories with good standardisation or under research conditions (regular external standard, duplicating analyses of every sample).

A potential weakness was lack of incorporation of alcohol into the equations. Alcohol depresses serum/plasma freezing point, increasing directly measured osmolality, with minimal impact on electrolytes, urea and glucose (increasing the difference between directly measured osmolality and osmolarity equations, the osmolar gap). The full Khajuria and Krahn^47^ equation incorporates blood alcohol:
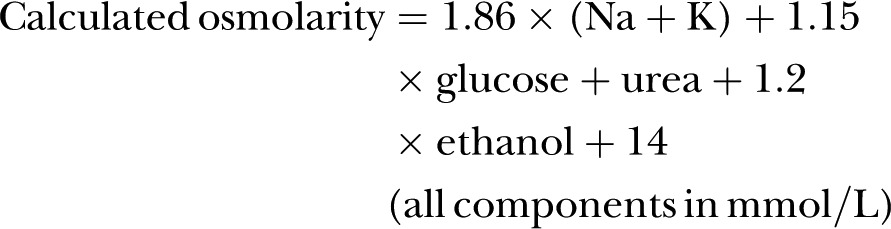


No participants in any cohort were noticeably inebriated, so we estimated effects on calculated osmolarity of drinking 0.5 or 1 bottle of wine 2 or 8 h before phlebotomy (assuming 70 kg body weight). Blood alcohol was negligible to 2.8 mmol/L^i^, highest in those drinking a bottle of wine 2 h before phlebotomy which increased calculated osmolarity by 3.4 mOsm/L. This alters the relationship between osmolarity and directly measured osmolality by only a small amount. Our analyses suggested that equations’ performance were slightly poorer in people more likely to have taken recent alcohol (figure 1), but this effect was not dramatic (and categories poorly defined). Despite likely alcohol intake in some participants osmolarity equations were good at screening for dehydration.

A further potential weakness was that we assumed that plasma osmolality (directly measured in Fortes) and serum osmolality (directly measured in the other cohorts) were equivalent. However, a high-quality study comparing lithium heparin plasma (as used in Fortes) with serum samples found equivalent directly measured osmolality.^48^ They also confirm that directly measured serum osmolality of fresh and frozen serum samples (stored at −78°C and thawed rapidly) were equivalent (although not when stored at higher temperatures).^48^ ^49^ On this basis we believe it is appropriate to use directly measured heparinised plasma osmolality and serum osmolality, as well as the osmolality of fresh and frozen samples (NU-AGE samples were the only frozen samples and were stored at −80°C), interchangeably. Previous studies have suggested a rise of 1–2 mOsm/kg in directly measured serum osmolality of samples stored at room temperature for up to 4 h before serum separation (as occurred in DRIE, and is standard practice for samples taken in primary care and transported to hospital laboratories for analysis).^50^

In all of our cohorts sodium and potassium concentrations were determined by indirect ISE (typically used in the large chemistry analysers in clinical laboratories, indirect ISE measures sodium on a plasma or serum sample that has been diluted with a large volume of diluent while direct ISE measures the electrolyte activity in the plasma water using a non-diluted whole-blood, plasma or serum sample and is typically used in point-of-care analysers).^51^ Indirect ISE (unlike direct ISE) is affected by the volume of non-aqueous cell components, lipids and proteins. While samples with high levels of lipid or protein are routinely re-analysed using direct ISE, low protein concentrations, common in older adults, may lead to higher sodium and potassium readings (compared to direct ISE),^52^ affecting 25% of intensive care unit samples and 8% of general hospital samples. While this may be reflected in some imprecision in the matching of osmolarity formulae to directly measured osmolality our results suggest that despite use of indirect ISE the Khajuria and Krahn equation is useful in screening for dehydration in older people.

The Khajuria and Krahn equation is generalisable across healthy free-living older people, frailer people living in residential care, and older people visiting emergency care or staying in hospital. It worked well in those with and without good renal function, with and without diabetes, with and without dehydration, with and without a tendency to drink alcohol and in men and women. It even worked well in patients with decompensated liver cirrhosis (who experience difficulty with sodium and water balance due to abnormalities in antidiuretic hormone and aldosterone, reflected in low mean sodium). It works well using standard hospital equipment for analysis of sodium, potassium, urea and glucose, in plasma and serum samples, in fresh and frozen samples.

Classical thought is that hypernatraemia principally explains raised osmolality when fluids are restricted,^53^ and the correlations in online supplementary table S2 were highest between serum osmolality and sodium, but there were also significant correlations with potassium, urea, creatinine and glucose in some cohorts, suggesting that these also contribute. The statistical significance of correlations in different cohorts may partially relate to cohort characteristics—we are more likely to see weak but statistically significant relationships in the larger cohorts (NU-AGE and DRIE). In NU-AGE there was no correlation between glucose and serum osmolality, but only 1% of NU-AGE participants had raised serum glucose, while in DRIE, where 20% of participants had raised serum glucose, the relationship with osmolality was weak but statistically significant. Raised serum sodium was not equivalent to raised serum osmolality in these cohorts of older people (only 4% had serum sodium >145 mmol/L, while 19% had serum osmolality >300 mOsm/kg, see online supplementary table S3). This reflects data in young fit army volunteers dehydrated by walking and fluid restriction in a hot environment, where only one of 36 volunteers who were clearly dehydrated (fluid loss assessed by weight loss) had raised serum sodium—plasma osmolality, with a threshold of 301 mOsm/kg, reflected hydration status much better than serum sodium.^25^ It appears that to assess hydration status plasma or serum osmolality is key, and to estimate serum osmolality well in cohorts with a variety of characteristics the contribution of sodium, potassium, glucose and urea are all crucial.

The finding that the Khajuria and Krahn^47^ equation (equation 32) was most useful in older people is consistent with our findings in DRIE data alone,^1^ but the equation may also be useful in younger populations. Heavens *et al*^54^ recently assessed a similar set of osmolarity equations against directly measured plasma osmolality in 60 young volunteers (aged 19–46 years), and found that five equations were useful, including Khajuria and Krahn's.^47^ The suggestion that this formula may be useful in young fit adults as well as in older adults adds weight to its utility.

**Figure 3:**
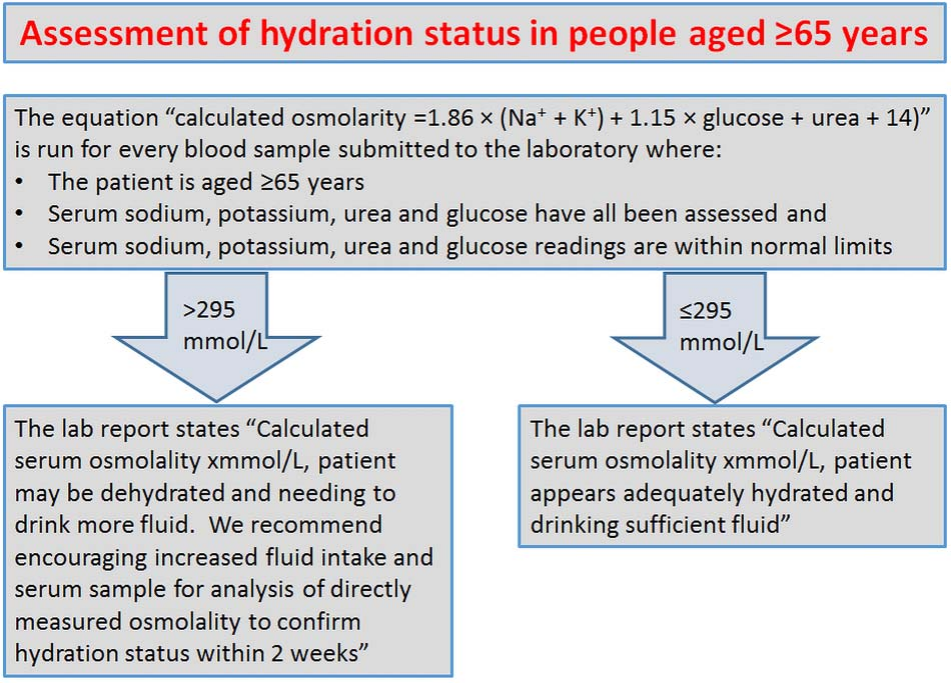
Suggested proforma for opportunistic assessment of hydration status by health laboratories.

Pathology laboratories could use this equation to report calculated osmolarity and hydration status of older people when analysing any blood sample including sodium, potassium, urea and glucose. For such routine screening for dehydration we could choose a cutpoint of ≥295 mOsm/L to provide sensitivity of 85% and specificity of 59%, where a positive finding could be followed up by directly measured serum osmolality to confirm diagnosis and rule out false positives (suggested proforma in figure 3). A finding of dehydration would be followed by consideration of glucose—if glucose is raised then treatment would address serum glucose (across our populations 16% of those with raised directly measured serum osmolality had raised glucose). Where glucose is not raised treatment would focus on increasing fluid intake. This screening would allow for early identification of dehydration (but not hypovolaemia) in older people, at little additional cost to the National Health Service. The alternative would be to assess for dehydration by directly measuring serum osmolality across the older population, but the cost of this, in laboratory time, resource and expense would be prohibitive without additional resources.

Using routine blood tests in older people to screen for dehydration using the Khajuria and Krahn formula for serum osmolarity would enable healthcare professionals and carers to provide appropriate support in older people by increasing fluid intake and improving and maintaining good hydration and thereby prevent associated poor health. This information could be provided automatically on the reports from pathology laboratories where serum sodium, potassium, urea and glucose have been measured, although to improve sensitivity (though increasing costs) positive results from this screening could be followed by assessment of directly measured serum osmolality. To assess the cost-effectiveness of these different screening models we need detailed data on the costs of dehydration and of serum osmolality analysis.

## Footnotes

i http://www.csgnetwork.com/bloodalcolevelcalc.html
